# Commentary: Maintenance of CD8^+^ T Memory Lymphocytes in the Spleen but Not in the Bone Marrow Is Dependent on Proliferation

**DOI:** 10.3389/fimmu.2018.00122

**Published:** 2018-02-01

**Authors:** Francesca Di Rosa, Benedita Rocha

**Affiliations:** ^1^Institute of Molecular Biology and Pathology, Consiglio Nazionale delle Ricerche, c/o Department of Molecular Medicine, Sapienza University, Rome, Italy; ^2^Population Biology Unit, CNRS URA 196, Institut Pasteur, Paris, France; ^3^INSERM, U1151, CNRS, UMR8253, Institut Necker Enfants Malades, Université Paris Descartes, Paris, France

**Keywords:** immunological memory, CD8 T cells, bone marrow, homeostatic proliferation, Cyclophosphamide

A short communication by Radbruch’s lab in *Eur J Immunol* addresses the question of memory CD8 T lymphocyte proliferation in mouse bone marrow (BM), by using repeated injections of Cyclophosphamide (CyP) as a tool to gain information on cell division *in vivo* (1). The authors claim that memory CD8 T cells in the BM do not proliferate, based on the evidence that their numbers are not reduced at a single time point 3 days after stopping CyP treatment, that is expected to kill all dividing cells. Moreover, they claim that memory CD8 T cells are resident in the BM, based on the results of a combination treatment with CyP and FTY720, a S1P receptor modulator that blocks cell egress from lymphoid organs. In the same issue of *Eur J Immunol*, Nolte et al. commented the findings by Radbruch and coworkers, highlighting their novelty and potential implications for the clinic (2). In this commentary, we would like to offer a different perspective, from a point of view that includes previous kinetics issues on CD8 T cell renewal in the BM and on the homeostatic regulation of memory T cells after an insult.

We propose that the lack of depletion of BM memory CD8 T cells by CyP is due to replacement by cells dividing in the BM so that, after a while, the numbers are again normal. Since only one time point was analyzed after CyP treatment, it is possible that cell number decrease went undetected in the BM, but not in the spleen, due to more extensive postdepletion proliferation and earlier recovery of memory CD8 T cells in the BM. This putative diversity between BM and spleen would echo the observed difference between the two organs when measuring the kinetics of variation of total nucleated cells, and of B cells, following mouse treatment with cytotoxic drugs (3).

Our interpretation is based on data from several laboratories, including our own, that memory CD8 T cells survive better and divide much faster than naïve CD8 T lymphocytes (4), that the homeostatic T cell division occurring after acute lymphodepletion tends to occur mostly in the BM, and that the BM dominates also in the case of poly:IC-induced CD8 T cell proliferation. For example, Silvestri’s lab showed that in non-human primates the percentage of Ki67^+^ T cells briskly increased in the BM during the recovery phase after acute Ab-mediated CD4 and CD8 T cell depletion (5). Skirecki et al. reported that in a mouse model of sepsis with lymphopenia, effector memory CD4 and CD8 T cells more extensively proliferated in the BM than in the spleen during postsepsis T cell restoration (6). Work in Ahmed’s lab (7) and in Di Rosa’s lab (8) showed that poly:IC-induced proliferation of memory CD8 T cells took place mostly in the BM and only to a lower extent in spleen and lymph nodes (LNs). Moreover, recent data from Rocha and colleagues showed that in naïve mice the number of CD44^high^ CD8 T cells specific for gp33 antigen was higher in the BM than in spleen and LNs (9). It should be noted that these cells were found in the BM of specific pathogen-free mice, never exposed to native gp33 carried by LCMV, suggesting that they were either cross-reactive or memory-phenotype CD8 T cells developed through homeostatic proliferation (9–11). Taken together, these data support the concept that BM is implicated in homeostatic division and accumulation of memory CD8 T cells under different perturbed conditions, including the recovery phase after drug-induced lymphodepletion.

Our interpretation can also apply to a previous paper by Radbruch and coworkers (12), in which they showed that BromodeoxyUridine (BrdU), a thymidine analog commonly used to measure *in vivo* proliferation, induced an abnormal expansion of memory CD8 T cells, especially in the BM. Although this effect had not been observed before in similar studies (7, 13, 14), a plausible explanation for the mechanism was not offered at the time, except saying that proliferation was Myd88-dependent (15). We already discussed the possibility that this abnormal expansion was related to the use of a higher dose of BrdU than that used by other labs (16, 17) and/or to BrdU contamination with LPS (18). When used at a high dose/high frequency of injection, BrdU is toxic to cycling cells inducing lymphodepletion. In these circumstances, homeostatic division should follow. A similar positive feedback loop has been observed in the case of HSC after BrdU treatment (19).

Furthermore, several evidence indicate that CyP has pleiotropic effects, even when low non-myeloablative doses of CyP are used (20). For example, 3 days after a single CyP injection a transient minor decrease of total BM cells was observed, followed by a subsequent increase peaking at day 7 (21). Some unexplained results by Siracusa and colleagues in the *Eur J Immunol* short communication point to CyP pleiotropism. They reported a 60% decrease of splenic B cells (1), even if the vast majority of peripheral B cells in the spleen are not in cycle (22). This CyP-induced effect likely reflects the elimination of B cell precursors in the BM, followed by the death and/or mobilization of splenic B cells, without sufficient replacement from BM compartment (23). We would like to particularly highlight some possible CyP indirect effects on CD8 T cells that were not taken into consideration. CyP induces type I IFN that in turn can regulate CD8 T cell homeostatic proliferation (24) and inhibit Treg cells (20, 25), possibly unleashing memory CD8 T cells from the Treg-mediated enforcement of their quiescent state (2, 26).

In brief, it appears that CyP is not the best method to assess *in vivo* lymphocyte proliferation. Memory CD8 T cell proliferation has been studied already by other methods by several groups, and results are all in agreement [references in Ref. (16)], except for the set of experiments with high doses of BrdU mentioned above (12). Analysis of untreated mice by DNA content assay consistently showed in different labs that the vast majority of memory CD8 T cells were in a quiescent state. At a snapshot in time, the frequency of dividing cells in S-G2-M phase of cell cycle was only 0.32–0.47% in the BM, still this low percentage was three to eight times higher than that in the spleen. In agreement with these data, CFSE and BrdU-labeling studies performed under controlled non-toxic conditions (14) documented that memory CD8 T cells proliferated in the BM to a higher extent than in spleen and LNs. The proportion of proliferating cells in the BM was not exiguous when labeling was performed over a few days. For example, in a 3-day BrdU experiment, BM CD44^high^ memory-phenotype CD8 T cells comprised 13% BrdU^+^ cells (13), while in a 7-day CFSE experiment they comprised 27% CFSE^low^ cells (27). Results were similar with antigen-specific memory CD8 T cells induced by intentional immunization [see full list of references in Ref. (16)].

Finally, Radbruch and colleagues used FTY720 in combination with CyP to rule out the possibility that memory CD8 T cells killed by CyP were replaced by incoming cells from the blood, thus resulting in a normal BM CD8 T cell number. They observed that a similar number of BM OVA-specific CD8 T cells was found in mice treated with CyP plus FTY720 and in mice treated with CyP alone and interpreted these results as evidence of stable residency of memory CD8 T cells in the BM (1). We already discussed above the possibility that memory CD8 T cells killed by CyP were replaced by *in situ* proliferating cells. Regarding the lack of FTY720 effects, our interpretation is that in the steady-state BM CD8 T cell number is regulated by equal cell entry and exit, while upon FTY720 treatment both types of exchanges with blood are shut down, so that BM CD8 T cell number does not change. This possibility is in agreement with the reported inhibitory effect of FTY720 on CD8 T cell egress from BM (28) and with data of BrdU pulse and chase (7), *in situ* labeling (29), and parabiosis experiments (30), all showing that BM CD8 T cells are in equilibrium with recirculating CD8 T cells in blood.

We predict that the undiscovered peculiarities of BM memory CD8 T cells will continue to fascinate “aficionados” in the field and also interest experts from other areas, attracted by the underlying biological questions plus the enormous translational potential, e.g., for vaccination, immunotherapy of cancer, etc. We look forward new data, interpretations, and debates.

## Author Contributions

FD and BR contributed with expertise on BM T cells and on T and B lymphocyte homeostasis, respectively, and wrote together the manuscript.

## Conflict of Interest Statement

The authors declare that the research was conducted in the absence of any commercial or financial relationships that could be construed as a potential conflict of interest.
